# Predictors for achieving adequate antenatal care visits during pregnancy: a cross-sectional study in rural Northwest Rwanda

**DOI:** 10.1186/s12884-023-05384-0

**Published:** 2023-01-26

**Authors:** Theogene Dusingizimana, Thiagarajah Ramilan, Janet L. Weber, Per Ole Iversen, Maurice Mugabowindekwe, Jeannine Ahishakiye, Louise Brough

**Affiliations:** 1 Department of Food Science and Technology, College of Agriculture, Animal Sciences and Veterinary Medicine, University of Rwanda, P.O. Box 210, Musanze, Rwanda; 2 School of Agriculture and Environment, Massey University, Tennent Drive, Palmerston North, 4442 New Zealand; 3 School of Food and Advanced Technology, Massey University, Tennent Drive, Palmerston North, 4442 New Zealand; 4 Department of Nutrition, Institute of Basic Medical Sciences, University of Oslo, Oslo, 0317 Norway; 5 Department of Haematology, Oslo University Hospital, Oslo, 0424 Norway; 6 Division of Human Nutrition, Faculty of Medicine and Health Sciences, Stellenbosch University, Tygerberg, 7505 South Africa; 7 Department of Geosciences and Natural Resource Management, University of Copenhagen, Copenhagen, Denmark; 8 Centre for Geographic Information Systems and Remote Sensing, College of Science and Technology, University of Rwanda, P.0. Box 3900, Kigali, Rwanda; 9 Human Nutrition and Dietetics Department, College of Medicine and Health Sciences, University of Rwanda, P.O. Box 3286, Kigali, Rwanda

**Keywords:** Antenatal care, Pregnancy, Rwanda

## Abstract

**Background:**

Inadequate antenatal care (ANC) in low-income countries has been identified as a risk factor for poor pregnancy outcome. While many countries, including Rwanda, have near universal ANC coverage, a significant proportion of pregnant women do not achieve the recommended regimen of four ANC visits. The present study aimed to explore the factors associated with achieving the recommendation, with an emphasis on the distance from household to health facilities.

**Methods:**

A geo-referenced cross-sectional study was conducted in Rutsiro district, Western province of Rwanda with 360 randomly selected women. Multiple logistic regression analysis including adjusted odd ratio (aOR) were performed to identify factors associated with achieving the recommended four ANC visits.

**Results:**

The majority (65.3%) of women had less than four ANC visits during pregnancy. We found a significant and negative association between distance from household to health facility and achieving the recommended four ANC visits. As the distance increased by 1 km, the odds of achieving the four ANC visits decreased by 19% (aOR = 0.81, *P* = 0.024). The odds of achieving the recommended four ANC visits were nearly two times higher among mothers with secondary education compared with mothers with primary education or less (aOR = 1.90, *P* = 0.038). In addition, mothers who responded that their household members always seek health care when necessary had 1.7 times higher odds of achieving four ANC visits compared with those who responded as unable to seek health care (aOR = 1.7, *P* = 0.041). Furthermore, mothers from poor households had 2.1 times lower odds of achieving four ANC visits than mothers from slightly better-off households (aOR = 2.1, *P* = 0.028).

**Conclusions:**

Findings from the present study suggest that, in Rutsiro district, travel distance to health facility, coupled with socio-economic constraints, including low education and poverty can make it difficult for pregnant women to achieve the recommended ANC regimen. Innovative strategies are needed to decrease distance by bringing ANC services closer to pregnant women and to enhance ANC seeking behaviour. Interventions should also focus on supporting women to attain at least secondary education level as well as to improve the household socioeconomic status of pregnant women, with a particular focus on women from poor households.

**Supplementary Information:**

The online version contains supplementary material available at 10.1186/s12884-023-05384-0.

## Introduction

Antenatal care (ANC) services are regarded as an important entry point to improve maternal and offspring health outcomes. ANC attendance provide health care providers with an opportunity to identify and address illnesses or early signs of obstetric complications. Through ANC, mothers receive counselling on maternal nutrition, infant and young child feeding practices, and are encouraged to deliver at a health facility, all of which give children an increased chance of survival and a better start to life. Research shows that settings with low availability and use of ANC services have high maternal mortality rates [1]. Investigation of case studies performed in Africa [2] and confidential enquiries into maternal deaths in low-income countries have identified the lack of ANC as a risk factor for poor pregnancy outcomes [3, 4].

Evidence also showed that ANC attendance facilitates timely management and improves outcomes for the most frequent complications (e.g. infection, pregnancy-induced hypertensive disorders and severe bleeding) associated with maternal morbidity and mortality in low-income countries [5]. Recent systematic reviews and a meta-analysis found that utilization of ANC from a skilled provider during pregnancy reduces the risk of neonatal mortality in low – and middle-income countries (LMICs), specifically in sub-Saharan Africa [1, 6, 7].

Globally, while nearly 90% of pregnant women access ANC with a skilled health personnel at least once, only three in five receive the WHO recommendation of at least four ANC visits [8]. The prevalence is even less in countries with the highest maternal and neonatal mortality rates such as in Sub-Saharan Africa, where only 53% pregnant women had at least four ANC visits in 2021 [8]. This situation suggests that a lot is still to be done to improve the quality of and or access to ANC in Sub-Saharan African countries.

In 2003, Rwanda adopted the WHO recommendation of a minimum of four ANC visits, with the first visit initiated during the first 12 weeks of gestation [9, 10]. The Rwandan Government has also implemented several strategies to promote ANC attendance and utilization. For example, in 2007 a community health work outreach program was launched in Rwanda as an essential strategy to support maternal health before and after pregnancy [11]. In addition, in 2010, the ANC care system in Rwanda was restructured so that the majority of ANC services are provided at community health centers [12]. Moreover, Rwanda’s maternal health strategy has put in place a health center incentivization scheme, which rewards health facilities based on the number of mothers whose first ANC contact occurs before 16 weeks of gestation and complete at least four ANC visits [13]. Despite these efforts, the number of women achieving the recommended four ANC visits remains low. While 98% of pregnant women contact their ANC provider at least once [14], recent data from the National Demographic and Health Surveys show that the increase in the proportion of pregnant women achieving at least four recommended ANC visits has been slow, from 35% in 2010 [15] to 47% in 2019/20 [14]. Recent data from Rwanda shows that nearly a half (42%) of pregnant women start seeking antenatal care after four months of pregnancy [14]. The WHO ANC guidelines recommends providing counselling to pregnant women on healthy eating and keeping physically active, educating women in undernourished populations on increasing energy and protein intake and encouraging pregnant women to take supplements such as folic acid and iron [16]. Low and/or late ANC attendance thus represents a missed opportunity for improving maternal and child health [17].

A systematic review of predictors of low ANC utilization in LMICs found that low educational attainment, decreased household income, unemployment, and higher cost of ANC are among factors associated with low ANC utilization [18]. In addition to demographic and socioeconomic factors, studies in LMICs have demonstrated that distance from health care facilities negatively correlates with utilization of primary health care services [19, 20]. However, some studies conducted in Kenya and Zambia found that distance did not always correlate with use of ANC services [21, 22]; highlighting the need for further research on the relationship between distance and use of ANC services in various contexts and settings.

Previous studies conducted in Rwanda on factors associated with ANC utilization have often focused on demographic and socioeconomic factors. These studies have identified several barriers to ANC utilization in Rwanda, including cultural norms, negative experiences with ANC providers, such as being criticized for registering for ANC too early or too late or presenting without a male partner [23]. Using secondary data, one study also examined the relationship between perceived distance and ANC access and/or utilization in Rwanda [24]. Although the results of that study suggested that physical distance from the user to the health facility is an important barrier to ANC utilization in Rwanda, the study used perceived distance rather than measured distance.

To our knowledge, no study has assessed the relationship between measured distance to health facility and utilisation of ANC services in Rwanda. We therefore wanted to explore factors associated with achieving the recommended four ANC visits in Rutsiro district, Northwest Rwanda, with a special emphasis on measured distance between households and the nearest health facility. Our hypothesis was that the longer the distance the lower the likelihoods to achieve the four recommended ANC visits.

## Methods

### Study settings

The present study was conducted in Rutsiro district (Supplementary file 1), one of the districts in Northwest Rwanda. Rutsiro has one district hospital, 18 health centers, and 36 health posts that served the population of 324, 654 in 2017 [25]. All maternal and child health services are provided by these health facilities, which are all, except two upgraded health posts (known as second-generation health posts), government owned (District Health Office, personal communication). Based on the Rwanda Demographic and Health Survey (RDHS), Rutsiro district performs poorly on child health, with 29% infant mortality rate in the ten-year period preceding RDHS 2019/20, and 44.4% stunting (short for child’s age) among children under 5 years of age [14]. Access to health services is also challenging in Rutsiro district due to long travel distances as a result of the nature of district’s landscape which hinders investments in transport infrastructure [26]. According to national survey data, the average perceived travel time to the nearest health facility in Rutsiro district is estimated to be more than 90 min, compared to the national average perceived travel time of about 60 min [27].

### Study design and participants

The data used in this study were collected as part of a cross-sectional survey conducted between September 2018 and January 2019 to investigate factors associated with child stunting in Rutsiro district. Details on sample size estimation and participant selection for the main cross-sectional study are described in detail elsewhere [28]. Briefly, the district was first divided into 3 zones based on main road network connecting the district to its neighbouring districts. Then, 18 villages were randomly selected (6 villages from each zone). In each of these villages, monthly growth monitoring lists were obtained from community health workers and used to compile a sampling frame from which participants were randomly selected. Eleven mothers who refused to participate or were not found in their homes were replaced by selecting the next name on the list. Mothers were eligible if (1) they had a child aged 6–23 months; (2) the child had no overt signs of illness; and (3) the mother was in the two lowest socioeconomic categories (out of four categories based on the Rwandan Government classification [29]). Of the 400 selected participants, 40 (10%) were excluded from the analysis due to missing data. Analysis was thus performed on 360 participants with completed data.

### Data collection

The household data was collected using a structured pre-tested questionnaire pre-programmed on the CommCare platform [30] and data were digitally captured using a tablet. The survey was conducted in the participants’ homes through face-to-face interview.

### Study variables

The outcome variable of our analysis was achieving the recommended four ANC visits, which was a binary choice variable defined based on the number of ANC visits achieved during pregnancy with the reference child. Four or more ANC visits was coded as 1, less than four visits was coded as 0.

Our independent variable of interest was the distance from household to the nearest health center. GPS coordinates for the location of both the households and health centres were recorded using a handheld Geographic Position System (GPS) device (Tremble Juno SB Handheld), allowing measurement of the distance from households to the nearest health centers. The distance (crow fly) was calculated using ArcGIS software (version 10.4), with an assumption that a household accesses the nearest health center. The calculations resulted into 360 routes connecting the 360 households to the closest health facilities.

Other independent variables included respondent and household characteristics. Respondent characteristics included age (coded as < 24 years, 25–29 years, ≥ 30 years), education level (primary education or less, some secondary education), marital status (coded as single/divorced/widowed, married/living with partner), and child’s birth order of the reference child (coded as first child, second child, third child and above). Household characteristics included socioeconomic group (coded as poor (lowest socioeconomic category) and slightly better-off (second lowest socioeconomic category), based on the Rwandan government classification [29]), household hunger level (coded as no /little hunger, moderate/severe hunger based on the Household Hunger Scale (HHS) following Ballard et al.’s methodology [31]), household size (continuous variable), number of children under 5 years of age (continuous variable). The HHS is an indicator of household hunger that is calculated by asking questions as to whether or not a specific condition associated with the experience of food insecurity ever occurred during the previous 4 weeks (30 days). We also included in our analysis three variables related to how respondents interacted with health services during pregnancy. The first was whether the respondent was a beneficiary of a supplementary food program during pregnancy (yes = 1, no = 0). The Government of Rwanda provides free fortified blended supplementary foods to pregnant and lactating women from households in the lowest socioeconomic quartiles [32]. The second was whether all household members were covered by a community health insurance (yes = 1, no = 0). The third was response to the question “do you and other household members always seek health care when necessary” (yes = 1, no = 0). Given that even households with community health insurance may not always seek health care due to additional costs associated to travel [33], information on whether respondent household’s members always seek health care when necessary was considered for analysis.

### Statistical analysis

Binary logistic regression was performed to assess independent associations between the outcome variable and the exposure variable of interest (i.e., distance – crow fly – from household to the nearest health facility), as well as individual predictor variables. Multiple logistic regression analysis was applied to determine the influence of distance and other independent variables on achieving four ANC visits represented as dichotomous outcome. All variables were considered as potential predictors and, thus, were included in the model. The magnitude of the effect of the independent variables associated with achieving the recommended four ANC visits was assessed through adjusted odds ratios (aOR). The significance of association was assessed through the corresponding 95% confidence intervals (CI). All analyses were performed using Statistical Package of Social Sciences (SPSS) version 25 (IBM).

## Results

The distance (crow fly) between maternal home and health center ranged from 0.02 km to 5.00 km, with a mean distance of 2.25 km (Table 1). The majority (65.3%) of women had less than four ANC visits during pregnancy. Eighty-three percent of women had primary education or less; 16% of women had some secondary education. Sixty-two percent of women were from households where all members were covered with community health insurance. Also 66% of the women reported that their household members seek health care when necessary. About three-quarters (73.5%) of the women were from slightly better-off households and 56.4% were from households with little to no hunger level. Only 11% of women benefited from the food supplementation program during pregnancy.

**Table 1:** Characteristics of study participants (*N* = 360)

Variables	Mean (min, max) or n (%)
Distance (crow fly; km)	Mean 2.25 km (0.02 km–5.00 km)
ANC attendance	
0–2	98 (27.2)
3	137 (38.1)
4 +	125 (34.7)
Marital status	
Single/widowed/divorced	70 (19.4)
Married/living with partner	290 (80.6)
Maternal age group (years)	
< 24	104 (28.9)
25–29	92 (25.6)
≥ 30	164 (45.6)
Child birth order	
1^st^ birth	117 (32.5)
2^nd^ birth	93 (25.8)
3^rd^ and above	150 (41.7)
Maternal education level	
Primary education or less	299 (83.1)
Some secondary education	61 (16.9)
Household size	4 (2, 11)
Number of children under 5	1 (1, 4)
Possession of community health insurance	
No	134 (37.2)
Yes	226 (62.8)
Household members seek health care when necessary	
No	124 (34.4)
Yes	236 (65.6)
Household hunger level	
Little or no hunger	203 (56.4)
Moderate or severe	157 (43.6)
Benefited from food supplementation program during pregnancy	
No	320 (88.9)
Yes	40 (11.1)
Household socioeconomic status	
Poor (Socioeconomic category 1)	94 (26.1)
Slightly better-off (Socioeconomic category 2)	266 (73.9)

Results from binary logistic regression analysis showed that increasing distance to the nearest health facility, marital status, maternal education level, household size, whether household members seek health care when necessary, and household socio-economic status were significantly associated with achieving the recommended four ANC visits (Table 2). Some of these factors were not significant in multivariable logistic regression analysis.

**Table 2:** Factors associated with achieving the recommended four ANC visits (*N* = 360)

Variables	cOR (95% CI)	*P*-value	aOR (95% CI)	*P*-value
Distance (km)	**0.84 (O.70, 0.99)**	**0.042**	**0.81 (0.67, 0.97)**	**0.024**
Marital status		0.033		0.23
Single/widowed/divorced	Ref		Ref	
Married/partner	1.87 (1.05, 3.34)		1.51 (0.77, 2.95)	
Maternal age group (years)		0.09		0.70
< 24	Ref		Ref	
25–29	1.18 (0.68, 2.07)		0.96 (0.48, 1.89)	
≥ 30	0.68 (0.41, 1.14)		0.72 (0.31, 1.68)	
Child birth order		0.13		0.48
1^st^ birth	Ref		Ref	
2^nd^ birth	1.07 (0.62, 1.84)		1.53 (0.76, 3.09)	
3^rd^ and above	0.66 (0.40, 1.09)		1.28 (0.49, 3.39)	
Maternal education level		0.007		**0.038**
Primary education or less	Ref		Ref	
Some secondary education	2.01 (1.23, 3.71)		1.90 (1.04, 3.47)	
Household size (continuous)	0.87 (0.77, 0.99)	0.036	0.99 (0.82, 1.19)	0.87
Number of children under 5 (continuous)	0.66 (0.43, 1.00)	0.051	0.70 (0.42, 1.18)	0.18
Household members always seek health care when necessary		0.016		**0.041**
No	Ref		Ref	
Yes	1.77 (1.11, 2.83)		1.70 (1.02, 2.81)	
Household hunger		0.094		0.85
Little or no hunger	Ref		Ref	
Moderate or severe	0.68 (0.45, 1.07)		0.95 (0.58, 1.57)	
Benefited from food supplementation program during pregnancy		0.47		0.12
No	Ref		Ref	
Yes	1.27 (0.66, 2.44)		1.88 (0.85, 4.14)	
Household socioeconomic status		0.013		**0.028**
Poor (Socioeconomic category 1)	Ref		Ref	
Slightly better off (Socioeconomic category 2)	1.93 (1.15, 3.25)		2.13 (1.09, 4.17)	

*cOR* Crude odds ratio, *aOR* Adjusted odds ratio, *CI* Confidence interval, *ANC* Antenatal care

In multiple logistic regression, we found a significant and negative association between distance and achieving the recommended four ANC visits. As the distance increases by 1 km, the odds of achieving four ANC visits decreased by 19% (aOR = 0.81, *P* = 0.024). Other significant variables found to be associated with achieving the recommended four ANC visits include education level, whether household members seek health care when necessary, and household socio-economic status. The odds of achieving the recommended four ANC visits were nearly two times higher among mothers with some secondary education compared with mothers with primary education or less (aOR = 1.90, *P* = 0.038). In addition, mothers who responded that their household members always seek health care when necessary had 1.7 times higher odds of achieving four ANC visits compared with those who responded otherwise (aOR = 1.7, *P* = 0.041). Furthermore, mothers from better-off households had 2.1 times higher odds of achieving four ANC visits than mothers from poor households (aOR = 2.1, *P* = 0.028).

## Discussion

Findings from the present study indicate that the factors significantly associated with achieving the recommended four ANC visits in Rutsiro district, include distance (crow fly) to health facility, maternal education level, health care seeking by household members, when necessary, as well as household socio-economic status.

Consistent with our findings, distance of household from health facility has been previously reported as one of the major challenges to accessing health care in different African countries. Studies show that the use of primary health care facilities decreases with increasing physical distance between patients and the health facility [34–36]. Using data from Rwanda Demographic Survey 2010, Manzi et al. also found that mothers who perceived distance as a barrier to accessing health facilities were more likely to delay ANC visits than mothers who did not [24]. Huerta Munoz and Källestål [37] also studied the geographical accessibility and spatial coverage of primary health facilities in the Western Province of Rwanda under three different scenarios (i.e. walking, walking and cycling, and walking and public transportation) by which the population access health care facilities. These researchers found that, under the three scenarios examined, the majority of the population does not have access (determined by distance and time) to the existing health facilities, and that access was the lowest under the walking scenario.

Based on the method used in the present study, the maximum estimated distance to the health facility was 5 km. However, the actual distances are undoubtedly greater than distances estimated in the present study due to the nature of landscape in the district. Like much of Rwanda, the landscape in Rutsiro district is mountainous, with an altitude as high as 3000 m above sea level [28], which may be compounded by the poor quality of roads [25]. Thus, given that walking is the primary mode of travel for the majority of Rwandans, the observed negative association between travel distance and achieving the recommended four ANC visits may point to the difficulties faced by pregnant women in Rutsiro district to reach health care facilities. This calls for strategies to facilitate the population, especially pregnant women in Rutsiro district, to access health facilities. As noted above, several strategies have been put in place in Rwanda to facilitate access to health services, including decentralization of community health services. More recently, the Government of Rwanda adopted a private–public partnership model to establish health posts at village level to facilitate access of health services, including ANC services [38, 39]. The model is regarded as an innovative strategy with potential to increase accessibility of health services, including ANC services, by decreasing the distance people have to travel to reach health facilities [39]. Before the health posts model is scaled-up, additional strategies to deliver and incentivize women should be explored. One such strategy is the use of mobile health clinics. For example the global health organization, i.e., Partners in Health, has used mobile clinics to deliver healthcare in remote villages in the Northern province of Rwanda [40]. Mobile clinics have also been used in Rwanda to deliver vaccines during the COVID-19 pandemic [41]. The Mobile clinics have been found to be an acceptable strategy to deliver and enhance coverage of ANC services in different countries such as The Gambia [42] and India [43], although research highlighted the need to pay attention on the quality of service provided through this model [44].

Our finding of a positive association between maternal education and achieving the recommended four ANC visits aligns with prior studies in low- and middle-income countries [18, 45]. It is well-known that mothers with higher level of education are also more likely to use health care services [46]. Maternal education is recognized as a key health resource that benefits the health of mothers and their children because educated mothers are more knowledgeable and more likely to understand the benefits of seeking health care services than mothers with a low level of education [47]. This underscores the need to increase the formal educational level of women in Rwanda and to explore educational opportunities for women with primary education or lower. Evidence shows that Rwanda has achieved remarkable success in increasing access to education, with a net primary school enrolment standing at 97% [48]; however, the majority of the children finish primary education with insufficient literacy and numeracy skills [49] and this can hinder women with only primary education level from taking advantage of or assimilating health messages received through different media platforms. Supporting women to attain at least secondary education level may have the potential to enhance uptake of ANC services in Rutsiro district.

Our results on the association between socioeconomic status and achieving the recommended ANC visits are consistent with previous studies across sub-Saharan Africa, which have shown that socioeconomic status is associated with maternal health care use [50, 51]. In a qualitative study on barriers and solutions for timely initiation of ANC in the Rwanda’s capital city, Kigali, poverty and challenges to related to health insurance were found to be among barriers to initiating ANC [33]. In 1999/2000, Rwanda put in place a social health protection system which consists of community-based health insurance (CBHI), also known as *Mutuelle de Santé,* to improve financial access to health services, including pre- and postnatal care services [52]. While women covered by the CBHI do not pay for attending ANC services, families still incur secondary costs such as costs for travelling (which may increase when a pregnant woman is accompanied), co-payments, or opportunity costs from missed days of work [33, 53]. Given that these costs increase with the number of ANC visits, they can represent a significant barrier to achieving the recommended ANC regimen. This calls for strategies to improve the household socioeconomic status of pregnant women in Rutsiro district, with a particular focus on those from poor households.

In the multivariate regression there is a positive association between participants’ report that their household members always seek health care when necessary and achieving the recommended ANC visits. In this association, health care seeking is likely a proxy for a number of factors which have been shown to be associated with seeking health care both in general and antenatal care. For example, in Rwanda, having full health care coverage is associated with health care utilization [54] and although ANC visits are free [23], Påfs et. al. found that some women had the perception that health insurance was also needed for ANC visits or procedures [23]. In addition, household resources can facilitate access to health care including ANC, e.g. support within the family [55, 56], or community networks [57]. Engagement with the health system has been suggested as a facilitator for access [57] and perceived lack of quality care or lack of respect from health professionals reported as a barrier [55, 57, 58]. Qualitative research needs to be carried out to fully understand the relevance of these factors in the study district and Rwanda in general. Addressing them will encourage use of health care by all, including ANC visits.

### Strengths and limitations

Strengths of the current study include its use of measured distance to examine the relationship between distance and ANC utilization. There are a number of limitations in this study. Firstly, the present study leveraged on availability of data from the main cross-sectional study, and this did not permit us to explore other factors such as intra-household dynamics (e.g. women’s resource control and bargaining skills) which may be associated with achieving the recommended four ANC visits in the studied district. Secondly, we acknowledge that straight-line distances used in the present study may not accurately capture the actual distance travelled by pregnant women to reach health facilities as people rarely reach their destinations along a straight line [59]. However, research has shown that straight-line distances are reasonable proxies for assessing physical accessibility of health services [60, 61]. Additionally, several studies have found that Euclidean distances and the distances obtained using complex methodologies such as road-network analysis are highly correlated, and researchers argued that substituting one for the other is unlikely to have substantial impact on analytical results [61–64]. Nevertheless, we recommend further validation studies using advanced techniques to determine travel distances and to assess the relationship between distance and attendance to ANC service. Thirdly, common to cross-sectional studies, the reliance on maternal recall may be prone to bias.

## Conclusion

Findings from the present study show that travel distances to health facilities, coupled with socio-economic constraints, including low education and poverty are associated with not achieving the recommended ANC regimen. These findings suggest the need for innovative strategies to decrease distance by bringing ANC services closer to pregnant women and to enhance their ANC seeking behaviours. Interventions should also focus on supporting women to attain at least secondary education level as well as improving the household socioeconomic status of pregnant women, with a particular focus on women from poor households.

## Supplementary Information

**Additional file 1: Supplementary file 1.** The study area map illustrating the location of the surveyed households as well the corresponding nearest health facilities in Rutsiro district in Rwanda.

## Data Availability

The data that support the findings of the current study are available from the corresponding author on reasonable request.
