# Supplementary material for: Predictors for achieving adequate antenatal care visits during pregnancy: a cross-sectional study in rural Northwest Rwanda
Source: BMC Pregnancy Childbirth. 2023 Jan 26;23:69. doi: 10.1186/s12884-023-05384-0 (PMC9878946; doi:10.1186/s12884-023-05384-0)
Supplement: Supplementary file 1 — Additional file 1: Supplementary file 1. The study area map illustrating the location of the surveyed households as well the corresponding nearest health facilities in Rutsiro district in Rwanda. [file 12884_2023_5384_MOESM1_ESM.docx]

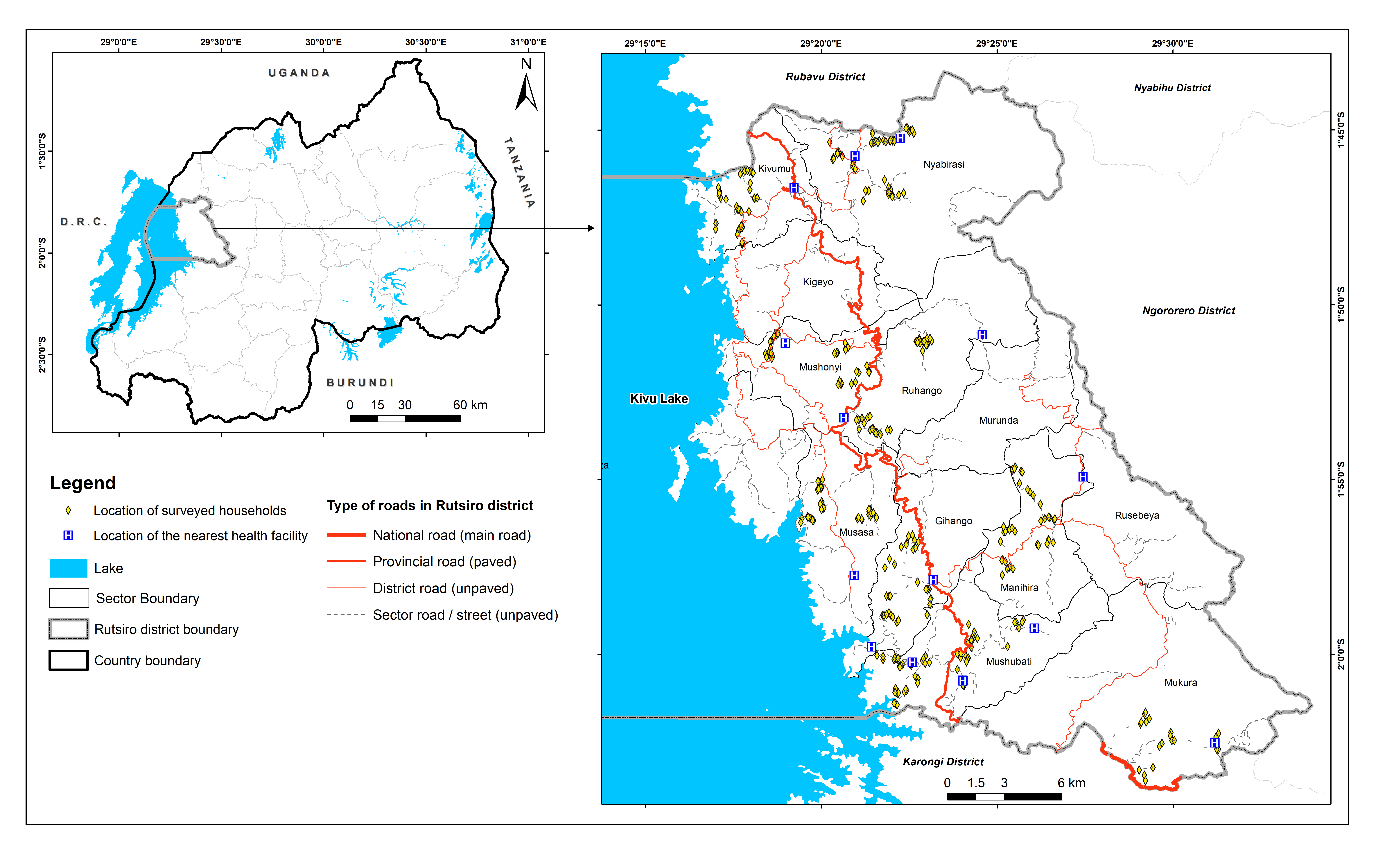


Supplementary file 1: The study area map illustrating the location of the surveyed households as well the corresponding nearest health facilities in Rutsiro district in Rwanda
